# FBG-Based Soft System for Assisted Epidural Anesthesia: Design Optimization and Clinical Assessment

**DOI:** 10.3390/bios12080645

**Published:** 2022-08-16

**Authors:** Francesca De Tommasi, Chiara Romano, Daniela Lo Presti, Carlo Massaroni, Massimiliano Carassiti, Emiliano Schena

**Affiliations:** 1 Unit of Measurements and Biomedical Instrumentation, Università Campus Bio-Medico di Roma, Via Alvaro del Portillo, 00128 Rome, Italy; 2 Unit of Anesthesia, Intensive Care and Pain Management, Università Campus Bio-Medico di Roma, Via Alvaro del Portillo, 00128 Rome, Italy

**Keywords:** epidural anesthesia, fiber Bragg grating sensor, force measurements, instrumented syringe, LOR detection, soft sensor

## Abstract

Fiber Bragg grating sensors (FBGs) are considered a valid sensing solution for a variety of medical applications. The last decade witnessed the exploitation of these sensors in applications ranging from minimally invasive surgery to biomechanics and monitoring physiological parameters. Recently, preliminary studies investigated the potential impact of FBGs in the management of epidural procedures by detecting when the needle reaches the epidural space with the loss of resistance (LOR) technique. In this article, we propose a soft and flexible FBG-based system capable of detecting the LOR, we optimized the solution by considering different designs and materials, and we assessed the feasibility of the optimized soft sensor (SS) in clinical settings. The proposed SS addresses some of the open challenges in the use of a sensing solution during epidural punctures: it has high sensitivity, it is non-invasive, the sensing element does not need to be inserted within the needle, and the clinician can follow the standard clinical practice. Our analysis highlights how the material and the design impact the system response, and thus its performance in this scenario. We also demonstrated the system’s feasibility of detecting the LOR during epidural procedures.

## 1. Introduction

Epidural anesthesia is broadly recognized as a loco-regional procedure extensively deployed to relieve pain by delivering a local anesthetic to the lower back in proximity to pain-transmitting nerves [1]. Wide-ranging clinical applications include childbirth, surgical procedure, and chronic back pain; one of the most common disorders worldwide with significant social impacts [2,3,4,5,6,7,8]. One of the critical aspects to achieve safe and effective epidural anesthesia is the accurate placement of the needle tip in the epidural space (ES) which is a small lumen (range in size by a few millimeters) lying near the spinal canal, in-between the dura mater and the ligamentum flavum [9]. During this procedure, the injection of the anesthetic within the ES is achieved by inserting the Tuohy needle through two lumbar vertebrae and delivering the drug by means of a catheter. The inadequate epidural block would lead to the risk of significant complications (e.g., dura mater perforation) caused by several reasons, including improper needle placement and secondary catheter displacement [10]. Generally, the anesthesiologist identifies the ES through the loss of resistance (LOR) technique based on the perceived perception of a drop in resistance resulting in the needle passage from a harder (i.e., ligamentum flavum) to a softer tissue (i.e., ES) [11]. The physician allows the needle advancement by pushing the syringe plunger containing saline solution or air. The pressure exerted during the penetration of different tissues (i.e., skin, fat, muscle) reaches a maximum value at the level of the ligamentum flavum. Once the epidural space is reached, a sudden decrease in pressure value occurs and should be felt by the clinician [12]. Therefore, the LOR success is strongly operator-dependent [13].

Several researchers promote the introduction of smart devices to support anesthesiologists during the procedure, allowing more accurate detection of the ES and reducing the risk of adverse events caused by a wrong procedure [14,15,16,17,18,19,20,21,22,23,24,25,26,27,28]. Some of these devices are commercially available and intended to provide visual and/or acoustic feedback to the operator as the ES is reached by measuring pressure during tissue crossing. In this case, the anesthesiologists are expected to spend a period of training to acquire knowledge of the equipment since some steps of the standard procedure change. Other investigations have proposed the use of diagnostic imaging techniques to increase procedure precision and guide needle placement. However, these solutions are space-consuming, overpriced, and rarely deployed in daily practice. Among non-commercial devices, the proposed solutions fall into the instrumentation of the plunger or the syringe needle using various technologies, mainly piezoresistive or fiber Bragg grating sensors (FBGs). All FBG-based solutions involve the placement of one or more sensors inside the Tuohy needle or attached to its surface to detect force or pressure experienced by the latter during the advancement. Nevertheless, such systems may impede fluid passage and contaminate the sterile field.

FBGs are rapidly growing in popularity over the last years in a variety of biomedical applications [21,22,24,25,26,29,30,31]. Indeed, several benefits advance FBGs over other technologies, including biocompatibility, reduced size, immunity to electromagnetic fields, inherent safety, and good metrological properties. In addition, FBG-based innovative systems can be encapsulated within silicone rubbers allowing a design close-fitting to the specific application and ensuring high flexibility and robustness [32,33,34]. As evidenced by scientific studies, the encapsulation of FBGs within silicone materials can impact the metrological characteristics of the entire developed system (e.g., in terms of sensitivity) since they are affected by the mechanical properties (e.g., Young’s modulus) of the selected material, the assigned geometry, and the interface bond between the sensor and the rubber coating [32,35,36]. Therefore, developing such smart devices requires an ad hoc design based on carefully selecting the silicone rubber, shape, and dimensions, which should be well-tailored to the specific application.

Recently, we proposed a novel soft and flexible system consisting of an FBG embedded within a flexible silicone rubber designed to fit the syringe plunger, thus allowing its instrumentation [25]. This study was an explorative assessment of a single system (made of Dragon Skin^TM^ 30, cylindrical in shape with a thickness of 8 mm) for LOR detection during epidural puncture. The findings obtained both in silico and in vivo support the promising idea and the capability of the system to support clinicians without disruption in the standard procedure, unlike previously proposed solutions. However, as pointed out above, the mechanical properties of specific silicone rubber and the thickness used to embed the sensor inside can influence the metrological properties of the overall system, thus affecting its output.

We investigated the right combination of materials and design to optimize the proposed soft system (SS). We quantitatively assessed the influence of two parameters related to material (three silicone rubbers with different stiffness) and design (three different thicknesses) by performing a metrological characterization of 9 SSs. This investigation allowed us to figure out how the outputs of the SS are affected by these parameters, thus selecting the most promising one. We evaluated the feasibility of the best suitable SS in clinical settings by relating its outcome to the clinician’s perception. We proved that our SS can correctly detect the LOR associated with entry into the epidural space and, therefore, has great potential for use in clinical settings.

## 2. Development of FBG-Based Soft Systems

This Section will describe the working principle of the SS designed to detect the LOR. In addition, we will give an overview of the design criteria, the materials used, and the manufacturing process carried out to develop the SSs, each consisting of an FBG embedded inside a flexible polymeric matrix. We tested SSs with different types of silicone rubber and thicknesses to evaluate the influence of both material and width on the metrological properties of the proposed solutions.

### 2.1. FBG Principle of Work

An FBG is characterized by periodic changes of the refractive index in the single-mode optical fiber core, resulting from the exposure to a variable pattern induced by a light source. Basically, FBG acts as a band-rejection filter. A reflection peak centered around a specific wavelength (i.e., λ_B_) satisfying the Bragg condition occurs because of a light signal crossing within the optical fiber, as highlighted in the following relationship [37]:

λ_B_ = 2⋅n_eff_⋅Λ (1)

where n_eff_ denotes the effective refractive index and Λ the spatial grating period.

FBGs are sensitive both to temperature and strain. Since these two parameters change, n_eff_ and Λ, λ_B_ undergo a shift (i.e., ∆λ_B_) upon variation of temperature or mechanical stress that can be expressed by the following equation [38]:

(2) $$∆\lambda_{B}=2\;\cdot \left[\Lambda \frac{\partial n_{\mathrm{eff}}}{\partial l}+{\;n}_{\mathrm{eff}}\frac{\partial \Lambda }{\partial l}\right]\;\varepsilon \;+2\;\left[\Lambda \frac{\partial n_{\mathrm{eff}}}{\partial T}+{\;n}_{\mathrm{eff}}\frac{\partial \Lambda }{\partial T}\right]\;\Delta T$$

The term $\left[\Lambda \frac{\partial n_{\mathrm{eff}}}{\partial l}+{\;n}_{\mathrm{eff}}\frac{\partial \Lambda }{\partial l}\right]\varepsilon$ refers to the effect of deformation (ε) on ∆λ_B_, corresponding to changes in n_eff_ and Λ. Meanwhile, $\left[\Lambda \frac{\partial n_{\mathrm{eff}}}{\partial T}+{\;n}_{\mathrm{eff}}\frac{\partial \Lambda }{\partial T}\right]\Delta$T represents the effect of temperature variation (ΔT) on the optical fiber. ∆λ_B_ occurs due to the thermal expansion phenomenon leading to alterations in the parameters (i.e., n_eff_ and Λ) affecting the Bragg condition. Equation (2) can also be expressed as follows:

∆λ_B_ = Sε⋅ε + S_T_⋅∆T (3)

where Sε is the strain sensitivity, generally equal to 1.2 pm/με, and S_T_ designates the temperature sensitivity approximately equal to 10 pm/ °C. By splitting the two contributions (ε and ∆T) causing the ∆λ_B_, it is possible to analyze the effect of ε and ∆T separately.

As detailed in [25], during epidural anesthesia, ∆λ_B_ appears because of the silicone rubber deformation by the anesthesiologist’s thumb (for tissues crossing) and its release once the epidural space has been reached, thus causing a shift on the right and on the left in the reflected spectrum, respectively. In this scenario, temperature contributions are considered negligible compared to the strain due to the force applied by the clinician to allow needle advancement.

### 2.2. Materials, Soft Systems Design, and Manufacturing Process

To develop the proposed SSs, we used three different silicone rubbers (i.e., Dragon Skin^TM^ 10,Medium, Dragon Skin^TM^ 20, and Dragon Skin^TM^ 30) from the same manufacturer (i.e., Smooth-On, Inc., Macungie, PA ). These rubbers are bi-component platinum silicones, exhibiting advantageous properties, such as biocompatibility, hard-wearing resistance, flexibility, and usage over a wide temperature range (−53 °C and 205 °C) [39]. Dragon Skin^TM^ 10, 20, and 30 differ in terms of Young’s modulus (E) (as provided by [40]) and curing time (expressed in hours -h-) specified in the technical bulletin. Table 1 below summarizes silicone rubbers’ characteristics.

As outlined, Dragon Skin^TM^ 10 is the most stretchable among the three materials, whereas Dragon Skin^TM^ 30 is the stiffer one. For each type of Dragon Skin^TM^ employed, we developed three types of SS, differing in the thickness of the rubber matrix and equal to 8 mm, 11 mm, and 14 mm, respectively. The use of material with variable stiffness and thickness allowed us to evaluate the influence of these mechanical and geometrical properties on the output of our systems.

**Figure 1:**
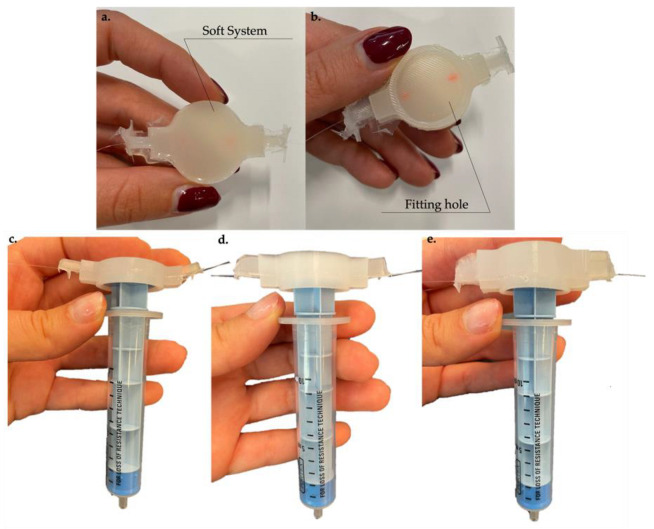
(**a**) One of the SSs developed. (**b**) SS mounting hole. (**c**) Arrangement on the LOR syringe of one of the 8 mm SSs developed. (**d**) Arrangement on the LOR syringe of one of the 11 mm SSs developed. (**e**) Arrangement on the LOR syringe of one of the 14 mm SSs developed.

As reported in [25], the design of the reported solution was conceived to fit the top of a syringe plunger. Each system is cylindrical in shape with a mounting hole to ensure perfect system adherence to the plunger upper part (see Figure 1a,b). The created hole allowed tight adhesion between the device and the plunger, thus preventing unwanted system movement during the procedure. Figure 1c–e shows the instrumented syringe by positioning the 8 mm, 11 mm, and 14 mm sensors on the plunger to highlight the difference among the SSs.

Three acrylate-coated FBGs (AtGrating Technologies, Shenzen, China) with a grating length of 10 mm, reflectivity values equal to 94%, and two different λ_B_, were used to develop the SSs, as detailed in Table 2.

**Figure 2:**
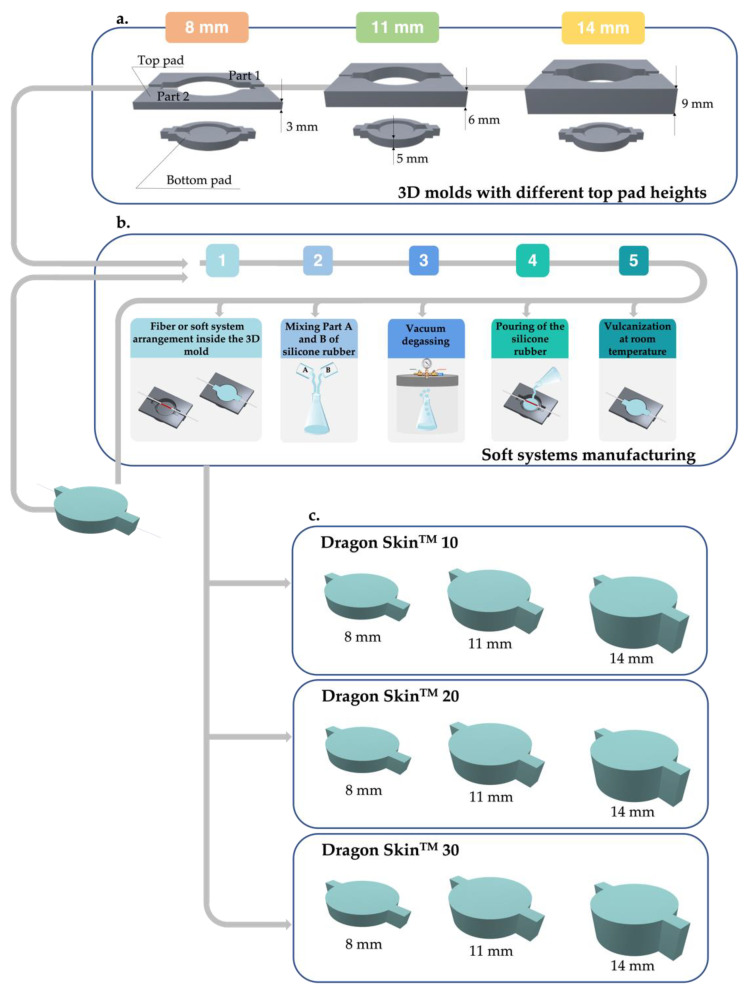
(**a**) Representation of 3D molds consisting of a bottom pad and top pad with different heights (i.e., 3 mm, 6 mm, and 9 mm) to gain the desired thicknesses (8 mm, 11 mm, and 14 mm). (**b**) Manufacturing steps: (1) fiber or soft systems arrangement inside the 3D mold; (2) mixing Part A and Part B of silicone rubber; (3) vacuum degassing; (4) pouring of the silicone rubber; (5) vulcanization at room temperature. (**c**) Extraction of 8 mm, 11 mm, and 14 mm SSs for Dragon Skin^TM^ 10, 20, and 30.

SSs were fashioned as desired by employing three 3D molds, whose design was detailed in our previous study [25]. Each mold consisted of two pads (i.e., top and bottom). The bottom pad (height of 5 mm) had a cylindrical cavity with a central insert to create the mounting hole. The top plate consisted of two parts to allow the FBG encapsulation. To gain the thickness variation of SSs, we realized different top pads of different heights (3 mm, 6 mm, and 9 mm), which were mounted on the bottom pad to achieve the desired thicknesses (8 mm, 11 mm, and 14 mm), as reported in Figure 2a. All molds implemented in a CAD environment (SolidWorks, Dassault Systems, Waltham, MA, USA) were realized in polylactic acid (PLA) using Ultimaker 2^+^ printer(Ultimaker, Utrecht, The Netherlands).

To obtain the SS with a thickness of 8 mm, we first used the mold with 3 mm top pads. The 10 mm long FBG (one of those detailed in Table 2) was placed in the middle of the bottom pad Figure 2(b1), and one of the silicone rubbers was mixed in its two components—Part A and Part B—as shown in Figure 2(b2), degassed to release air bubbles trapped inside as seen in Figure 2(b3), and poured, Figure 2(b4). After a curing time (varying depending on the Dragon Skin used) at room temperature, as evidenced in Figure 2(b5), the SS was safely removed from the mold. Once the 8 mm system was ready, it was metrologically characterized, and then we repeated the manufacturing process to obtain the following thickness. Therefore, the already developed system (instead of the single optical fiber) was arranged inside the bottom pad, attaching 6 mm top pads to achieve the 11 mm width. The other steps of the process remained unchanged. Likewise, it was conducted to obtain the 14 mm SS, except using the mold with 9 mm top pads. The process described was repeated two more times using the other two remaining types of Dragon Skin not employed in the previous round. Thus, for each of the three rubbers used (Dragon Skin^TM^ 10, 20, and 30), it was possible to obtain three different thicknesses of the proposed system (as evidenced in Figure 2c).

## 3. Metrological Characterization

This Section is devoted to describing the metrological characterization carried out for each developed SS to assess the influence of silicone rubber stiffness and thickness on the response to the force of the SSs. The first section (Section 3.1) concerns the description of the experimental set-up implemented to carry out the compression tests, the second section (Section 3.2) is dedicated to the data analysis, and the section (Section 3.3) to the results retrieved from the collected data.

### 3.1. Compression Tests

**Figure 3:**
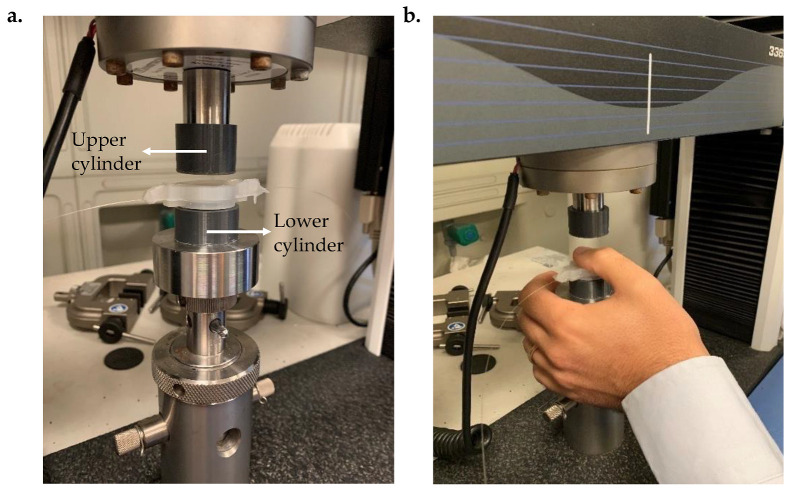
Configurations of the compression tests carried out for all SSs developed (for each Dragon Skin^TM^ and thickness) in the absence (SS accommodated between the two PLA cylinders) (**a**) and in the presence (**b**) of a volunteer thumb between the upper cylinder and the SS.

To evaluate the response of the nine SSs (differing in materials and thickness), a total of 36 compression tests were performed for each silicone rubber through a testing machine (model 3365, Instron^®^, Norwood, MA, USA). Specifically for the same Dragon Skin^TM^, 12 compression tests were carried out for each thickness under two distinct settings (six tests for each one): (i) the SS was placed between two PLA cylinders of the same diameter as the syringe plunger. This solution aimed at guaranteeing conditions closer to the real scenario than the experimental set-up adopted in [25]. The upper cylinder adhered perfectly to the machine indenter, while the other one was well positioned on the bottom plate using double-sided adhesive tape (as shown in Figure 3a); (ii) a volunteer inserted their thumb between the upper cylinder and the SS, thus covering the whole FBG sensitive length (as shown in Figure 3b). This approach allowed us to reproduce the real clinical scenario during which the physician’s thumb pushes on the syringe plunger to advance the needle through different tissue layers as much as possible.

In all trials, an external force (F), ranging between 0 N–35 N, was applied at a low displacement speed (i.e., 5 mm/min) to simulate quasi-static conditions. F values were measured by a load cell (full-scale value of 500 N and accuracy of ±0.25% of the reading value, serial number 69376, Instron^®^, Norwood, MA, USA) and acquired at a sampling rate of 100 Hz. Meanwhile, for each test, λ_B_ values from the SS were recorded at the same rate via an optical interrogator (si255, Hyperion Platform, Micron Optics Inc., Atlanta, GA, USA).

### 3.2. Data Analysis

Data were post-processed in a MATLAB^®^ environment to obtain the calibration curves (∆λ_B_ vs. F) per developed SSs (i.e., for each material and each thickness) both in the absence, T, and in the presence of thumb, PT. First, data collected from the testing machine (F values) and SS (∆λ_B_ values) were synchronized to gain the ∆λ_B_ trends as a function of applied F for each of the six tests performed in AT and PT. The related uncertainty was calculated through a Student’s t-distribution with five degrees of freedom and a confidence level of 95% for AT and PT settings. The ∆λ_B_ values were normalized (∆λ_Bn_) to the maximum ∆λ_B_ recorded among all the conditions to highlight the influence of materials and thicknesses on the systems’ response. Finally, the curves obtained (∆λ_Bn_ vs. F) were fitted using a linear model.

### 3.3. Results

**Figure 4:**
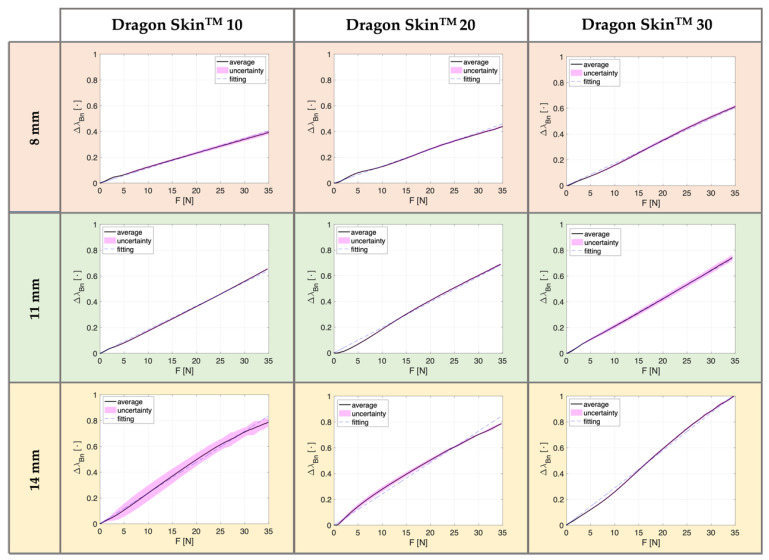
Normalized responses of the SSs (∆λ_Bn_ vs. F) obtained in AT. The three columns distinguish the curves obtained according to the material used (i.e., Dragon Skin^TM^ 10, 20, and 30), and the three rows marked with different colors refer to the thicknesses (8 mm in orange, 11 mm in green, and 14 mm in yellow). In each plot, the continuous black line represents the average ∆λ_Bn_ vs. F, the highlighted area in magenta the related uncertainty, and the dotted blue line the linear fitting.

**Figure 5:**
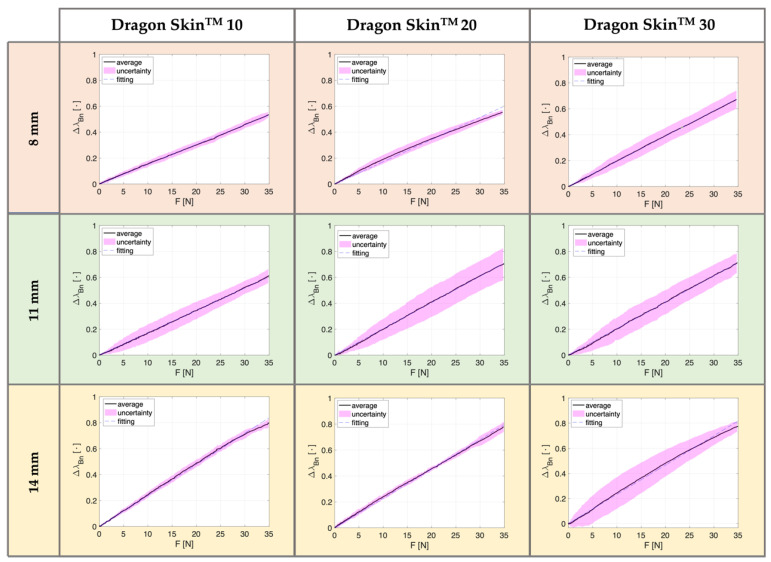
Normalized responses of the SSs (∆λ_Bn_ vs. F) obtained in PT. The three columns distinguish the curves obtained according to the material used (i.e., Dragon Skin^TM^ 10, 20, and 30), and the three rows marked with different colors refer to the thicknesses (8 mm in orange, 11 mm in green, and 14 mm in yellow). In each plot, the continuous black lines represent the average ∆λ_Bn_ vs. F, the highlighted area in magenta represent the related uncertainty, and the dotted blue line represent the linear fitting.

Figure 4 and Figure 5 compare the SSs’ response (∆λ_Bn_ vs. F) for the two reproduced settings—AT and PT—respectively. Each figure is organized in a matrix, in which the three columns classify the curves obtained according to the silicone rubbers employed (first column for Dragon Skin^TM^ 10, second for Dragon Skin^TM^ 20, and third for Dragon Skin^TM^ 30) and the three rows marked with orange, green, and yellow refer to SSs of 8 mm, 11 mm, and 14 mm thickness, separately. In every plot, the continuous black line denotes the average ∆λ_Bn_ vs. F trend across the performed six compression tests, the magenta area highlights the related uncertainty, and the dotted blue line reports the linear fitting. In both configurations (i.e., AT and PT), results showed a linear behavior for all the reproduced SSs (in terms of materials and thicknesses) with R^2^ values around 0.99 for all the eighteen curves retrieved. Table 3 summarizes the maximum percentage ∆λ_Bn_ recorded for all developed SSs at each configuration (AT and PT), the silicone rubber used (Dragon Skin^TM^ 10, 20, and 30), and thickness (8 mm, 11 mm, and 14 mm). We found that the ∆λ_Bn_ increased with thickness. This behavior was corroborated by both AT and PT compression tests, showing higher ∆λ_Bn_ values for 14 mm width SSs (the maximum value recorded for Dragon Skin^TM^ 30 in AT), thus also resulting in the highest sensitivity values.

Furthermore, in the case of AT, the maximum ∆λ_Bn_ values increased with the stiffness of the silicone rubber. Only in one case were the same values obtained for Dragon Skin^TM^ 10 and 20, with an SS of 14 mm in width (i.e., 79%, as evidenced in Table 3). However, this tendency was not confirmed for the metrological characterization performed in PT, whereby the increase in the maximum percentage ∆λ_Bn_ as the silicone rubber changes occurred only for SSs with thicknesses of 8 and 11 mm (see Table 3). Indeed, for the highest thickness (i.e., 14 mm), the maximum ∆λ_Bn_ values were comparable across the three Dragon Skin^TM^ (80%, 78%, and 77%, respectively). Moreover, compression tests carried out in AT conditions showed small uncertainty values corroborating the repeatability in the behavior of our proposed SSs (see Figure 4). Differently, the SSs’ response in PT exhibited higher uncertainty values because of different inter-test variability resulting from the thumb placement on the silicone rubber (see Figure 5).

The SS made of Dragon Skin^TM^ 30 with 14 mm of thickness showed the highest sensitivity. For this SS, we calculated the linearity error according to the following equations [41]:

u_L_(x) = y(x) − y_L_(x) (4)

(5) $$\%u_{\mathrm{Lmax}}=\frac{u_{\mathrm{Lmax}}}{r_{0}}\times 100$$

where u_L_(x) is the linearity error, y(x) represents the measured values, and y_L_(x) denotes the predicted output values obtained from the linear relationship. From u_L_(x), it is possible to determine the maximum linearity error in percentage (i.e., %u_Lmax_) as the ratio between the maximum u_L_(x) and the full-scale output (r_0_). The experimental results showed a %u_Lmax_ equal to 2.36%. This finding revealed that the proposed fitting is suitable for the specific scenario, considering that the main objective is to detect the sudden decrease in ∆λ_B_ when the needle crosses the ligamentum flavum and reaches the epidural space.

Given the outcomes obtained in terms of the highest sensitivity value (i.e., 0.058 nm⋅N^−1^ in absence of normalization) and the lower %u_Lmax_, this SS was selected to be used in clinical settings.

## 4. Clinical Assessment of the Optimized System on Patients Affected by Low Back Pain

In this section, we will describe the clinical assessment of the optimized system (in terms of material and thickness) carried out on four patients affected by low back pain who underwent the epidural procedure.

### 4.1. Experimental Set-Up

**Figure 6:**
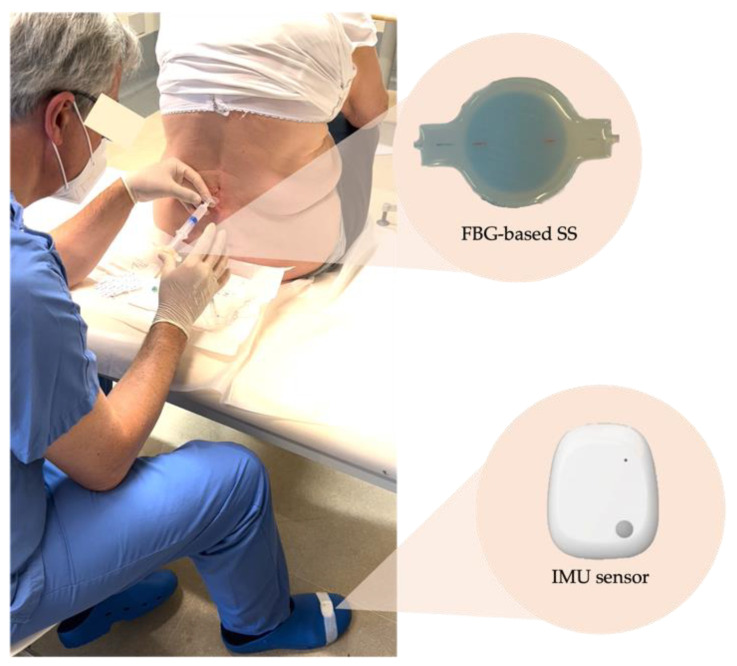
Experimental set-up of one of the epidural procedures performed.

After the metrological characterization, the selected SS (Dragon Skin^TM^ 30 with 14 mm of thickness) was validated in clinical settings. For this purpose, we enrolled four patients affected by lower back pain who came to the outpatient clinic of our hospital to perform an epidural puncture. The study carried out was approved by the Ethical Committee of our institution (Ref: 04.16-OSS). All the patients were recruited in conformity with the guidelines provided by the Declaration of Helsinki and consented to participation by signing the informed consent. The four epidural procedures were executed by the same anesthesiologist (M.C. with over 20 years of practice). In the first phase, the patient was instructed to take a fetal position on the treatment table to allow easy identification of the intervertebral space. At the same time, the clinician was responsible for sterile field preparation. Once the Tuohy needle was inserted in the puncture site (changeable according to the patient’s pathology), the clinician placed the SS on the syringe’s plunger (see Figure 6). The SS was connected to an optical interrogator (si255, Micron Optics Inc., Atlanta, GA, USA) to collect its output for the entire duration of the procedure at a sampling frequency of 1 kHz.

A single lightweight inertial measurement unit (IMU) sensor (MetaMotionS, MBIENTLAB Inc., San Francisco, CA, USA) was used to relate the clinician’s perception to the output of our system when the LOR occurred. The IMU sensor was placed on the physician’s foot with adhesive tape to collect 3-axis acceleration data (as shown in Figure 6). The coordinate system was such that the foot-to-head direction was for the z-axis, the lateral direction for the y-axis, and the dorso-ventral direction for the x-axis. Before starting, we simultaneously struck three times on both systems to synchronize data collected from the SS and the accelerometer. After positioning and for the entire procedure, the physician was asked to hold still and tap his foot when he felt the LOR. This resulted in a prominent peak along the three axes of the accelerometer, which could be used as ground truth for validating the LOR on the signal collected by the SS. Hence, the acceleration signal allowed for the assessment of whether the actual entry of the needle into the ES, perceived by the anesthesiologist and detected by the accelerometer, corresponds to a decrease in the ∆λ_B_ collected by the SS. Acceleration raw data were saved in the device’s internal memory and sampled at 100 Hz with a full-scale ±2 g and resolution of 16 bit.

### 4.2. Data Analysis

Data collected from the SS and IMU sensor were post-processed in a MATLAB^®^ environment. Firstly, ∆λ_B_ values and acceleration signals of the three axes (i.e., x, y, and z) were cut, considering as a reference the minimum value after the third peak related to the synchronization between the two devices. Later, signals from the SS were down-sampled at the same sampling frequency as the accelerometer signal (i.e., 100 Hz), and a moving average filter was used to reduce noise contributions.

From the acceleration values recorded along the three axes, we calculated the vector magnitude unit (VMU), as reported in the equation below:

(6) $$\mathrm{VMU}=\sqrt{x^{2}+{\;y}^{2}+{\;z}^{2}}$$

∆λ_B_ and VMU vectors were plotted as a function of time, only taking into account the stage related to the epidural procedure starting from the point at which the anesthesiologist pushed on the SS to allow Tuohy’s needle advancement. This phase was easily identified on the FBG signal because the physician was asked to push on the SS three times at the procedure’s beginning. In this way, it was possible to evaluate the time elapsed between the sudden drop in ∆λ_B_ and the perturbation in the VMU signal related to tapping of the clinician’s foot on the floor once the LOR is perceived.

### 4.3. Results

**Figure 7:**
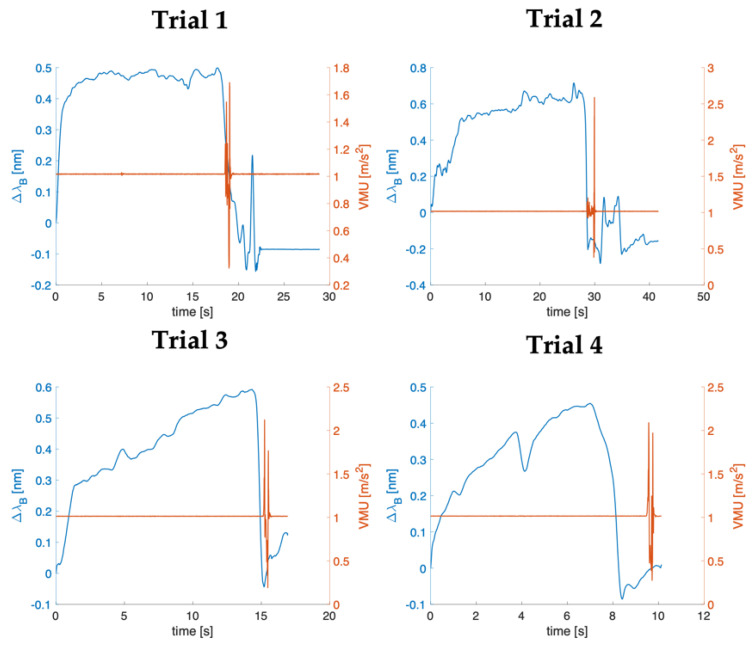
∆λ_B_ (blu lines) and VMU (orange lines) signals as a function of time expressed in s for each of the patients enrolled (i.e., Trial 1, 2, 3, and 4).

Figure 7 reports the ∆λ_B_ and VMU signals as a function of time in blue and orange, respectively, for each enrolled patient. All the trends collected from the SS showed similar trends that can be divided into three main phases: (i) a rapid increase in ∆λ_B_ due to the relevant forces applied by the clinician to advance the needle through several tissue layers, (ii) a phase when ∆λ_B_ values were kept above a threshold, which fluctuated depending on the force applied during the procedure, (iii) a sudden decrease in ∆λ_B_ when the needle tip crossed the ligamentum flavum and reached the epidural space. Further variations in ∆λ_B_ values after this step were attributable to the force applied by the operator on the plunger to inject the remaining liquid inside the syringe. As evidenced in Figure 7, the maximum ∆λ_B_ recorded ranged between 0.45 nm and 0.72 nm. In trial 4, a slight decrease in ∆λ_B_ before the LOR occurs. This was attributable to a re-positioning of the needle during the procedure.

VMU values calculated by the signals recorded from the accelerometers were approximately 1 g during the whole procedure, since the physician’s foot was fixed to the ground. VMU showed a sudden change due to the foot tap once the LOR is felt. As clearly revealed from the plots, the pronounced peak in VMU occurs shortly after the instantaneous drop in SS output, demonstrating the ability of SS to detect the LOR associated with the reaching of the epidural space. Indeed, the time between the ∆λ_B_ drop and the first changes in VMU values is always shorter than 1.5 s.

## 5. Discussion and Conclusions

In this study, we proposed a soft FBG-based system to detect the LOR during epidural procedures. The success of these treatments is strongly dependent on the operator’s expertise, which may be time-consuming and sometimes challenging. Therefore, over the years, the awareness of the need to develop technologies that can improve the procedure’s success and support physicians in its execution was raised. Here, we propose a solution designed to fit perfectly with the syringe’s plunger routinely used by the anesthesiologist. Our proposal possesses the advantage of non-alteration in the standard practice because it is intended to support the operators and not supersede them.

Compared to other FBG-based solutions, our SS is the first completely non-invasive approach. Indeed, approaches presented by [21,22,23,24] suggest instrumenting the Tuohy’s needle or replacing it with a new one. Scientific articles published by Carotenuto et al. [21,22] proposed inserting an FBG array inside the Tuohy needle to track pressure variations as it moves through several tissues. Differently, Ambatsha et al. [24] introduced the use of the custom-made system replacing the standard needle. It consisted of two cylinders made of steel connected by a band that housed in the middle a 3 mm-FBG. More recently, in [23], authors proposed using a shape-sensing guidance system, including four distributed optical fiber sensors attached to the external surface of the Tuohy needle. These proposals intend to include optical fibers inside the needle, involving the risk of occlusion in the anesthetic flow. In addition, these solutions (both inside the needle and attached to its surface) result in a high level of sterile field contamination and are not intended for prompt reuse as they require a careful sterilization process. They also need a mounting phase on the new needle (since the same needle cannot be used on another patient), which greatly dilates the treatment time. No less important, these solutions were designed as alternatives to the LOR method and demanded substantial changes in the traditional procedure because, in some cases, the physician cannot use the syringe plunger to advance the needle. Our system overcomes these previously listed limitations as it is re-usable, immune to sterile field contamination, and does not require any alterations in the standard settings.

Compared to the previous study [25], this study allowed us to provide additional knowledge concerning the influence of the geometry and mechanical properties of the silicone rubber on the SS’s response. For this purpose, we developed and tested 9 SSs differing in silicone rubber stiffness (i.e., Dragon Skin 10, 20, and 30) and thickness (i.e., 8 mm, 11 mm, and 14 mm). Specifically, for each of the listed materials, SSs were developed with the three thicknesses mentioned above. The influence of silicone rubbers and geometry was evaluated by carrying out a metrological characterization for each developed system in two different scenarios (with and without the presence of a volunteer’s thumb) and in a range of force between 0 N and 35 N. In the literature, F estimated during epidural procedures revealed a maximum value equal to 11.5 N [42]. This result derived from experiments carried out in an ex vivo animal model, which therefore differed from real conditions. For this reason, we decided to consider a maximum F of at least three times greater than the maximum reported in [42], to be sure of pinpointing the range of interest for the specific application. Results revealed that, for the same applied force, the FBG response changes significantly according to the SS employed, suggesting that the best SS is the one made of the stiffest material (i.e., Dragon Skin^TM^ 30) and the highest thickness (i.e., 14 mm). Indeed, this SS showed the highest output variation for the same applied force compared to all others developed, thus resulting in the greatest sensitivity value. This step was crucial for customizing the SS design to the specific application.

Moreover, to assess the capability of the optimized SS in LOR detection, we enrolled four patients who planned to undergo an epidural procedure performed by the same anesthesiologist. The response of the SS across the four trials showed the same behavior characterized by a sudden drop in ∆λ_B_ when the needle tip crossed the ligamentum flavum and reached the epidural space due to the LOR. Using an IMU sensor attached to the clinician’s foot allowed for a reference system. Indeed, the operator was instructed to tap his foot once he felt the LOR. In this way, it was possible to determine whether there was any correspondence between the clinician’s perception and the decrease in the ∆λ_B_ collected by the SS. Results obtained for the four trials showed that the pronounced peak due to foot movement on the acceleration signal occurred strictly at or shortly after the instantaneous drop in FBG output, proving that our solutions can correctly detect the LOR.

However, we are aware that our study is limited by the small number of subjects enrolled and the engagement of the same anesthesiologist who performed the procedures. For this reason, future studies will focus on increasing the sample size and enlisting practitioners with different expertise in the field to evaluate the proposed solution’s usability and viability under a wide range of working conditions. Moreover, consideration will be paid to real-time data processing through a dedicated graphical interface capable of providing feedback to the user once the ES is detected.

## Figures and Tables

**Table 1:** Dragon Skin^TM^ silicone rubber characteristics.

Silicone Rubber	E [MPa]	Curing Time [h]
Dragon Skin^TM^ 10	0.24	5
Dragon Skin^TM^ 20	0.47	4
Dragon Skin^TM^ 30	0.74	16

**Table 2:** Technical specifications of FBGs used.

Silicone Rubber	Coating	FBG Length [mm]	λ_B_ [nm]	Reflectivity Value [%]
Dragon Skin^TM^ 10	Acrylate	10	1540	94
Dragon Skin^TM^ 20	Acrylate	10	1540	94
Dragon Skin^TM^ 30	Acrylate	10	1536	94

**Table 3:** Maximum percentage ∆λ_Bn_ recorded for all developed SSs at each configuration (AT and PT), silicone rubber used (Dragon Skin^TM^ 10, 20, and 30), and thickness (8 mm, 11 mm, and 14 mm).

	Dragon Skin^TM^ 10		Dragon Skin^TM^ 20		Dragon Skin^TM^ 30	
	AT	PT	AT	PT	AT	PT
8 mm	39%	53%	44%	55%	61%	67%
11 mm	66%	61%	69%	70%	74%	71%
14 mm	79%	80%	79%	78%	100%	77%

## Data Availability

The data presented in this study are available on request from the corresponding author. The data are not publicly available due to privacy reasons.
